# Cryptic environmental conjugative plasmid recruits a novel hybrid transposon resulting in a new plasmid with higher dispersion potential

**DOI:** 10.1128/msphere.00252-24

**Published:** 2024-05-21

**Authors:** Iván Muñoz-Gutiérrez, Luis Cantu, Jack Shanahan, Miray Girguis, Marlene de la Cruz, Luis Mota-Bravo

**Affiliations:** 1 School of Biological Sciences, University of California, Irvine, California, USA; University of Wisconsin-Madison, Madison, Wisconsin, USA

**Keywords:** antibiotic resistance, criptic plasmid, transposons

## Abstract

Cryptic conjugative plasmids lack antibiotic-resistance genes (ARGs). These plasmids can capture ARGs from the vast pool of the environmental metagenome, but the mechanism to recruit ARGs remains to be elucidated. To investigate the recruitment of ARGs by a cryptic plasmid, we sequenced and conducted mating experiments with *Escherichia coli* SW4848 (collected from a lake) that has a cryptic IncX (IncX4) plasmid and an IncF (IncFII/IncFIIB) plasmid with five genes that confer resistance to aminoglycosides (*strA* and *strB*), sulfonamides (*sul2*), tetracycline [*tet*(*A*)], and trimethoprim (*dfrA5*). In a conjugation experiment, a novel hybrid Tn21/Tn1721 transposon of 22,570 bp (designated Tn7714) carrying the five ARG mobilized spontaneously from the IncF plasmid to the cryptic IncX plasmid. The IncF plasmid was found to be conjugative when it was electroporated into *E. coli* DH10B (without the IncX plasmid). Two parallel conjugations with the IncF and the new IncX (carrying the novel Tn7714 transposon) plasmids in two separate *E. coli* DH10B as donors and *E. coli* J53 as the recipient revealed that the conjugation rate of the new IncX plasmid (with the novel Tn7714 transposon and five ARGs) is more than two orders of magnitude larger than the IncF plasmid. For the first time, this study shows experimental evidence that cryptic environmental plasmids can capture and transfer transposons with ARGs to other bacteria, creating novel multidrug-resistant conjugative plasmids with higher dispersion potential.

**IMPORTANCE:**

Cryptic conjugative plasmids are extrachromosomal DNA molecules without antibiotic-resistance genes (ARGs). Environmental bacteria carrying cryptic plasmids with a high conjugation rate threaten public health because they can capture clinically relevant ARGs and rapidly spread them to pathogenic bacteria. However, the mechanism to recruit ARG by cryptic conjugative plasmids in environmental bacteria has not been observed experimentally. Here, we document the first translocation of a transposon with multiple clinically relevant ARGs to a cryptic environmental conjugative plasmid. The new multidrug-resistant conjugative plasmid has a conjugation rate that is two orders of magnitude higher than the original plasmid that carries the ARG (i.e., the new plasmid from the environment can spread ARG more than two orders of magnitude faster). Our work illustrates the importance of studying the mobilization of ARGs in environmental bacteria. It sheds light on how cryptic conjugative plasmids recruit ARGs, a phenomenon at the root of the antibiotic crisis.

## INTRODUCTION

Bacteria have evolved different mechanisms to survive toxic compounds for billions of years, including antibiotics produced by other microorganisms (1). While some bacteria produce antibiotics to inhibit the growth of competitors in their ecological niche, other bacteria acquire antibiotic resistance genes (ARGs) in an arms race to overcome the inhibition (2). Natural environments are a reservoir of ARGs and the ultimate source of the ARGs present in pathogenic bacteria in clinical settings (3–7). These ARGs can be mobilized horizontally between different bacteria by the mobile genetic elements (MGEs): transposons and conjugative plasmids (8).

Transposons are DNA segments able to move to new locations in the same or different DNA molecules (8). Inverted repeat (IR) sequences at each flank of the transposon delimit the area of DNA that moves during transposition (8). A transposase encoded in the transposon binds to the IRs and performs DNA translocation (8). After the transposition event, the target site used by the transposase to integrate the transposon is duplicated, creating direct repeats (DRs) right next to each IR (8). The presence of DRs flanking the transposon is the signature of a transposition event. In addition to the transposase gene, transposons can carry cargo genes such as ARGs (8).

Conjugative plasmids are self-transmissible extrachromosomal DNA molecules (8). The conjugation machinery encoded by the plasmid allows the transmission of the plasmid to other bacteria (8). The movement of antibiotic-resistant transposons to conjugative plasmids can increase ARG dispersion (8). Cryptic conjugative plasmids (without ARGs) from natural environments are a particular concern because they can recruit ARGs from the enormous environmental metagenome (9). Additionally, as described in the present report, cryptic conjugative plasmids can capture ARGs from other conjugative plasmids. The newly formed plasmids may disperse antibiotic resistance more efficiently because they are a hybrid between transposons containing multiple ARGs and a highly efficient conjugation apparatus of cryptic plasmids, thus expanding the diversity of molecules responsible for the evolution of antibiotic resistance in pathogenic bacteria. Hence, it is imperative to understand how cryptic conjugative plasmids from natural environments acquire ARGs.

Bioinformatics analysis shows the association between conjugative plasmids and transposons in disseminating ARGs between different bacteria species (10). Also, computational analysis shows the involvement of particular transposons in disseminating ARGs in different environments; for instance, there is a high association between the Tn3 family of transposons and different classes of ARGs in aquatic environments (11). Extensive documentation shows conjugative plasmids of clinically isolated bacteria carrying transposons with ARGs (8) and reports with cloning vectors demonstrated the transposition of ARGs between molecules (12–14). However, no information exists on how conjugative plasmids from natural environments acquire ARGs. Specifically, there is no record in the literature of cryptic conjugative plasmids from environmental bacteria acquiring ARGs due to the translocation of a transposon.

In the present work, we show the spontaneous transposition of ARGs from one plasmid into a cryptic conjugative plasmid from an environmental bacterium isolated from a lake in Baton Rouge, LA. The spontaneous translocation of the ARGs occurred during a conjugation experiment between the isolated *Escherichia coli* SW4848 (donor) and the lab strain *E. coli* DH10B (recipient). The novel 22,570 bp Tn7714 transposon of the Tn3 family translocated the ARGs from an IncF to an IncX cryptic conjugative plasmid. The new IncX multidrug-resistant plasmid has a conjugation frequency of two orders higher than the original IncF plasmid carrying the Tn7714 transposon. Our results illustrate that cryptic plasmids with high conjugation rates can capture transposons with ARGs, increasing the spread of ARGs.

## RESULTS

### A bacterium isolated from a lake in a city park carries clinically relevant ARGs

**Fig 1:**
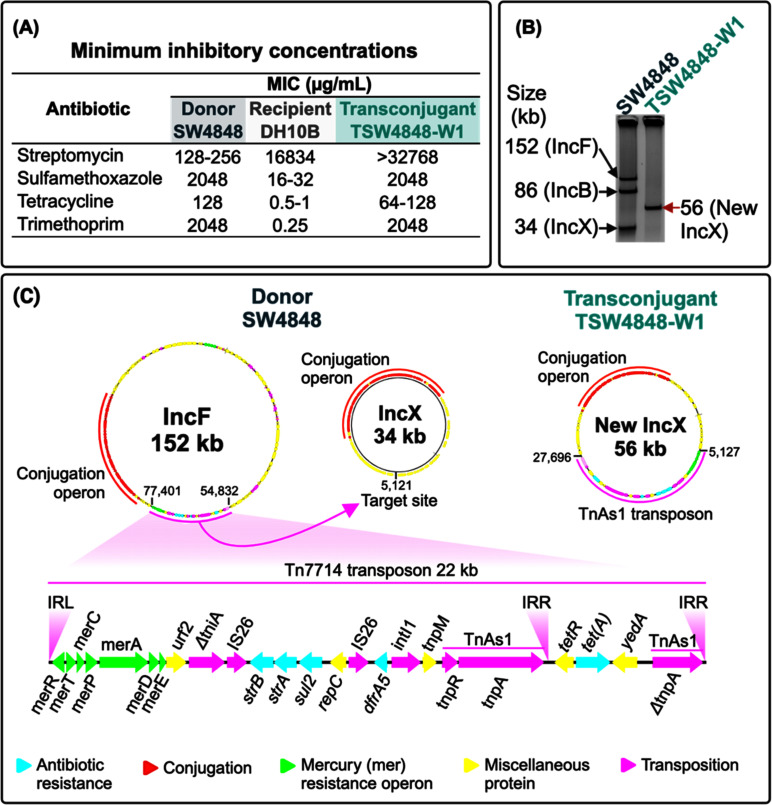
Translocation of Tn7714 into a cryptic conjugative plasmid during conjugation. (**A**) Antibiotic-resistance phenotype of *E. coli* SW4848 (donor), *E. coli* DH10B (recipient), and TSW4848-W1 (transconjugant). The antibiotics used were based on the ARGs found in the Tn7714 transposon in panel C. The MICs were repeated three times. (**B**) Whole plasmid gel electrophoresis of *E. coli* SW4848 and TSW4848-W; this picture was cropped from Fig S1 to show in detail only the large plasmids. *E. coli* SW4848 also has a small 3 kb plasmid. The sizes of the plasmids were obtained by whole-genome sequencing of *E. coli* SW4848 and TSW4848-W1. Lane two shows the new IncX plasmid that spontaneously formed by the translocation of the Tn7714 transposon from the IncF plasmid into the new IncX plasmid. (**C**) Description of the relevant plasmids of *E. coli* SW4848 and TSW4848-W1. The IncF and IncX plasmids belong to SW4848 (NZ_CP10 7285.1 and NZ_CP107287.1), and the new IncX plasmid belongs to TSW4848-W1 (NZ_CP110861.1). All these plasmids have genes coding for conjugation apparatus (red genes). The pink box shows the Tn7714 transposon’s main details. The Δ*tnpA* gene is a truncated version of the Tn7714 *tnpA* with an intact inverted repeat right (IRR). The Tn7714 transposon in the IncF plasmid is located between the 54,832 and 77,401 nucleotides and flanked by the 5′-AATTA-3′ DRs. The Tn7714 integrated into the IncX plasmid at the target site 5′-TATGTC-3′ located at nucleotide 5,121. In the new IncX plasmid, the 5′-TATGTC-3′ DRs flank the Tn7714 transposon between the 5,127 and 27,696 nucleotides.

During our bacterial analysis of water from natural environments in Baton Rouge, LA, we collected an isolate labeled SW4848 at the City Park Lake (30.429116 N 91.167173 W). Matrix-assisted laser desorption/ionization time-of-flight (MALDI-TOF) mass spectrometry identified SW4848 as an *E. coli*. Whole-genome sequence (GCA_025808215.1) revealed that SW4848 carries ARGs conferring resistance to aminoglycosides (*strA* and *strB*), sulfamethoxazole (*sul2*), tetracycline [*tet*(*A*)], and trimethoprim (*dfrA5*). Minimum inhibitory concentration (MIC) tests confirmed the resistance to the previously mentioned antibiotics (Fig. 1A).

*E. coli* SW4848 carries three large plasmids of 152 (NZ_CP107285.1), 86 (NZ_CP107288.1), and 34 kb (NZ_CP107287.1; Fig. 1B). In addition, SW4848 has a small 2.6 kb plasmid (NZ_CP107286.1; Fig. S1). These plasmids belong to the incompatibility types IncFII/IncFIIB, IncB/O/K/Z, IncX4, and Col440I, respectively. Figure 1C depicts the characteristics of the relevant plasmids for this study: the IncFII/IncFIIB and IncX4, and from here, we will refer to these plasmids as IncF and IncX, respectively.

In *E. coli* SW4848, all ARGs in SW4848 are on the IncF plasmid inside a novel hybrid Tn21/Tn1721 transposon of 22,570 bp (belonging to the Tn3 family) that we registered in the Transposon Registry database, and it was designated Tn7714 (Fig. 1C). In addition to the ARGs, the Tn7714 transposon has a mercury resistance operon (*mer* operon), a class 1 integron, and an IS26-composite transposon (Fig. 1C). The integron carries the *dfrA5* trimethoprim resistance gene. The IS26-composite transposon carries *strA*, *strB*, and *sul2*. Downstream of the *tnpA* transposase gene, there is *tet(A*) and a truncated version of *tnpA* with an intact inverted repeat right (IRR). Therefore, in addition to the inverted repeat left (IRL), the Tn7714 transposon has two IRRs (Fig. 1C). The presence of the DRs 5′-AATTA-3′ next to the IRL and the IRR of the truncated *tnpA*, flanking the 22 kb transposon, serves as a hallmark of the transposition of Tn7714 into the IncF plasmid (Fig. 1C).

### The environmental *E. coli* disseminates clinically relevant ARGs via conjugation

A conjugation experiment with the environmental *E. coli* SW4848 as the donor and the lab strain *E. coli* DH10B as the recipient resulted in the transconjugant *E. coli* TSW4848-W1 (Fig. 1A). The conjugation frequency was 1.74 × 10^−8^ transconjugants per donor (±1.2 × 10^−8^, *n* = 3). We picked one colony from the transconjugants’ plate to obtain MICs, gel electrophoresis, and whole-genome sequencing to confirm the transfer of the plasmid from the donor (SW4848) to the transconjugant (TSW4848-W1; Fig. 1A through C). The MIC results confirmed the movement of the ARGs from SW4848 into TSW4848-W1 because the transconjugant increased its MICs by orders of magnitude compared with DH10B (Fig. 1A).

### Cryptic conjugative plasmids can capture ARGs and disseminate the acquired genes

The whole plasmid gel electrophoresis analysis of SW4848 and the TSW4848-W1 transconjugant revealed an unexpected result: TSW4848-W1 harbors a 56 kb plasmid that does not match the size of any SW4848 plasmids (Fig. 1B). To understand the discrepancies in the results shown by the gel electrophoresis, we sequenced, assembled, and annotated the genome of the TSW4848-W1 transconjugant (GCA_026240775.1). Our computational analysis discovered the translocation of the Tn7714 transposon that mobilized from the IncF plasmid (Fig. 1C, left) to the cryptic IncX plasmid (Fig. 1C, center), creating the new IncX plasmid (Fig. 1C, right; NZ_CP110861.1). The 5′-TATGTC-3′ DRs flanking the transposon confirmed the translocation of Tn7714 into IncX. As shown in the whole plasmid gel electrophoresis of Fig. 1C, the sum of the Tn7714 transposon (22 kb) and the IncX plasmid (34 kb) matches the size of the new IncX plasmid (56 kb). The movement of the new plasmid into *E. coli* DH10B was possible because the IncX plasmid encodes all the genes for a conjugation apparatus (Fig. 1C, right).

### The conjugation rate of the new IncX plasmid is more than 100 times higher than the IncF plasmid

**Fig 2:**
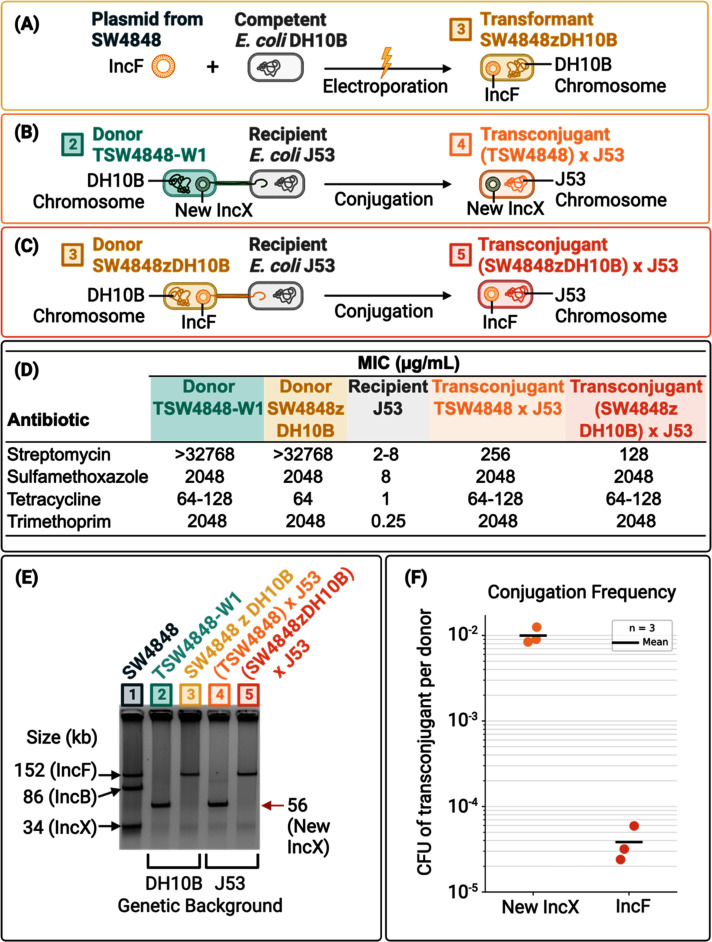
Experiments to compare the conjugation frequency of IncF and IncX plasmids. (**A**) Depiction of the transformation of *E. coli* DH10B with the IncF plasmid. We transformed *E. coli* DH10B with the IncF plasmid to have the IncF plasmid in the same genetic background as the new IncX. We used the transformant SW4848zDH10B to compare the conjugation frequency of the IncF vs the new IncX plasmid. (**B**) Conjugation depiction of *E. coli* TSW4848-W1 (donor) carrying the new IncX plasmid and *E. coli* J53 (recipient). (**C**) Conjugation depiction of SW4848zDH10B (donor) carrying the IncF plasmid and *E. coli* J53 (recipient). (**D**) Antibiotic-resistance phenotype of donors, recipients, and transconjugants depicted in panels B and C. The antibiotics used were based on the ARGs found in the Tn7714 transposon shown in Fig. 1C. (**E**) Plasmid phenotype of donors (bacteria with DH10B chromosome) and transconjugants (bacteria with J53 chromosome). Well 1 shows the plasmids of the environmental *E. coli* SW4848. The plasmid sizes of SW4848 were obtained by whole-genome sequencing. Well 2 shows the new IncX plasmid obtained during the conjugation of *E. col*i SW4848 (donor) and *E. coli* DH10B (recipient). The size of the new IncX plasmid (indicated by the red arrow) was obtained after whole-genome sequencing of TSW4848-W1. Well 3 shows that SW4848zDH10B has only the IncF plasmid. Wells 4 and 5 show the successful transfer of the IncF and new IncX plasmids from *E. coli* DH10B (donor) into *E. coli* J53 (recipient) during conjugation experiments (this picture was cropped from Fig S1 to show in detail only the large plasmids). (**F**) Conjugation frequency of the IncF and new IncX plasmid from *E. coli* DH10B (donor) into *E. coli* J53 (recipient). The plot shows the average conjugation frequency of three independent experiments.

To test whether the IncF plasmid is conjugative and to eliminate any potential interactions with other plasmids present in SW4848, we transformed the IncF plasmid into *E. coli* DH10B, creating the strain SW4848zDH10B (Fig. 2A). The whole plasmid gel electrophoresis of Fig. 2E, well 3, confirms that SW4848zDH10B carries the IncF plasmid. Additionally, as described in Fig. 2B and C, the new strain SW4848zDH10B allowed us to compare the conjugation frequency of the IncF vs the new IncX plasmid in the same genetic background, i.e., both strains have the DH10B chromosome. We carried parallel conjugations using SW4848zDH10B and TSW4848-W1 as donors and the lab strain *E. coli* J53 as the recipient (Fig. 2B and C). MIC tests showed that the conjugative plasmids successfully transferred all the ARGs into the transconjugants because all the transconjugants’ MICs increased orders of magnitude compared to the recipient J53 (Fig. 2D). Wells 4 and 5 of the gel electrophoresis in Fig. 2E confirmed the mobilization of the new IncX and IncF plasmids during the conjugation. Even though the IncF plasmid is conjugative, its conjugation frequency is two orders of magnitude lower than the new IncX frequency (Fig. 2F).

### The novel *E. coli* SW4848 Tn7714 transposon is a Tn21/Tn1721 hybrid

**Fig 3:**
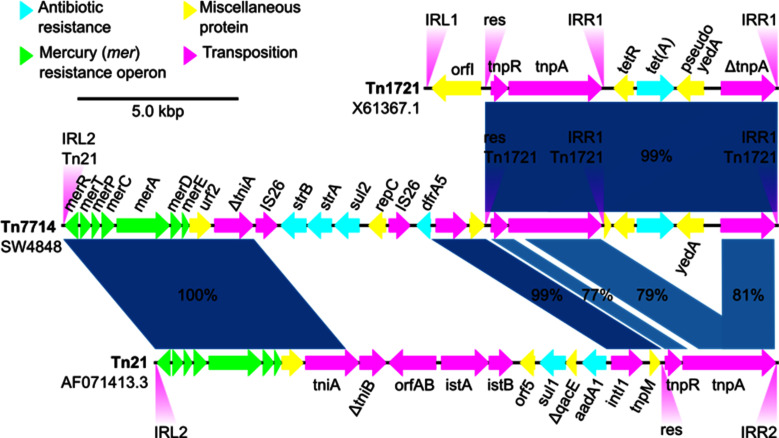
Alignment visualization of SW4848-Tn7714, Tn1721, and Tn21 transposons. The blue shading represents nucleotide identity, showing the conservation of different regions between the transposons. The percentages in the blue shading represent identity. The IRL2 of Tn7714 and Tn21 (AF071413.3 [18]) and the two IRR1s of Tn7714 and Tn1721 (X61367.1 [17]) are 100% identical. The nucleotide locations of the Tn7714’s IRs in the IncF plasmid are 77,401 (IRL2), 54,832 (IRR1), and 60,320 (IRR1). The resolution sites (res) of Tn7714 and Tn1721 are 99% identical. All the sequences are the reference transposons in TnCentral and the Transposon Registry.

To understand the origin of the novel SW4848 Tn7714 transposon, we performed a comparative analysis with the TnCentral and ISfinder databases (15, 16). Figure 3 shows an alignment visualization comparing the novel Tn7714 (middle sequence) with Tn1721(top sequence, X61367.1 [17]) and Tn21 (bottom sequence, AF071413.3 [18]). The alignment illustrates the identity (99%) of the resolvase (*tnpR*), transposase (*tnpA*), and tetracycline resistance [*tet*(*A*)] genes of the novel Tn7714 transposon and the Tn1721 transposon in the right side. While on the left side, the *mer* operon, *intI1*, and *tnpM* are identical between the novel Tn7714 and Tn21 transposons. Therefore, the novel *E. coli* SW4848 Tn7714 transposon is a hybrid between Tn21 and Tn1721 transposons.

## DISCUSSION

While the environmental microbiome is an enormous reservoir of ARGs (19), very little is known about the pool of cryptic plasmids with high conjugation rates that can rapidly disperse ARGs. This knowledge is critical because novel ARG-plasmids combinations can cross ecosystem compartments (environment-food-clinic) and species boundaries, threatening the entire health system (19). Here, we show how a novel multidrug-resistant Tn7714 transposon translocated from an IncF plasmid into an IncX cryptic plasmid. The new IncX plasmid has the conjugation apparatus of the original IncX plasmid plus the Tn7714 transposon with its cargo ARGs from the IncF plasmid. The new IncX plasmid has two orders of magnitude higher conjugation frequency than the IncF (Fig. 2F). Our results show, for the first time, experimental evidence that cryptic plasmids in environmental bacteria can capture and transfer ARGs to other bacteria, producing a more efficient multidrug-resistant conjugative plasmid.

The conjugation experiment with *E. coli* SW4848 as the donor (with the IncF and IncX plasmids) revealed that the Tn7714 transposition occurred in the donor strain before the new IncX plasmid was transferred to the recipient (*E. coli* DH10B), based on the following three observations: (i) the gel electrophoresis showed only the band of the new IncX plasmid in the transconjugant (*E. coli* TSW4848-W1; Fig. 1B), (ii) the whole-genome sequence of the transconjugant *E. coli* TSW4848-W1 did not show any segment of the of IncF plasmid (other than the transposon), and (iii) the new Tn7714 transposon found in the new IncX plasmid was flanked by DR, indicating a transposition event (DRs flanking the transposon is the signature of a transposition event).

The conjugation rate is affected by multiple factors, including the ratio of donors and recipients, the time of the mating, and the presence or absence of an antibiotic during conjugation (20, 21). Studies have used different experimental conditions to measure the conjugation rates in the laboratory. In this study, we used the most common experimental conditions used in the literature, and we report the conjugation rate as a transconjugants-to-donor ratio (21). We did not use antibiotics during the incubation period, and we washed and incubated the cells in 0.85% NaCl before the selection of transconjugants. We incubated all the mating experiments during the same period. For a review on the limitations of this method, particularly in relation to the problem of post-transfer selection, see references 20, 21.

Conjugation rates are also affected by the bacterium host chromosome because proteins that play a role in the conjugation mating pair stabilization may express differently between species and strains (22, 23); therefore, the conjugation rate of a single plasmid may differ when it is present in different bacteria strains. For this reason, we only compared the conjugation of the plasmids IncF and new IncX when they were in the same genetic background (chromosome of *E. coli* DH10B). Our results show that the conjugation rate of the IncF is two orders of magnitude lower than the new IncX conjugation rate when they are compared in the same genetic background (i.e., in *E. coli* DH10B; Fig. 2F).

The mobilization of the Tn7714 transposon from the environmental *E. coli* SW4848 (donor) to *E. coli* DH10B (recipient) requires two sequential steps: (i) the translocation of the Tn7714 transposon from the IncF plasmid to the cryptic IncX plasmid, and (ii) the conjugation of the new IncX plasmid (carrying the Tn7714 transposon) to the *E. coli* DH10B (recipient). The combined frequency of these two steps is 1.74 × 10^−8^ transconjugants per donor (SD of 1.2 × 10^−8^, *n* = 3). In contrast, the conjugation of the new IncX plasmid (carrying the Tn7714 transposon) from *E. coli* DH10B (donor) and *E. coli* J53 (recipient), without the translocation of the Tn7714 transposon, is 9.97 × 10^−3^ transconjugants per donor (SD of 2.21 × 10^−4^, *n* = 3). This result shows that once the multidrug-resistant Tn7714 transposon translocates to the new IncX plasmid, it can be dispersed at a much higher frequency than when it is located in the IncF plasmid in the original environmental *E. coli* SW4848. On the other hand, the conjugation of the IncF plasmid from *E. coli* DH10B (donor) and *E. coli* J53 (recipient) is 3.84 × 10^−5^ transconjugants per donor (SD of 1.85 × 10^−5^, *n* = 3; Fig. 2F). Since we could not obtain the IncF transconjugants using the environmental *E. coli* SW4848 as a donor, we had to electroporate this plasmid to *E. coli* DH10B; therefore, the conjugation rate of the IncF plasmid from environmental *E. coli* SW4848 (donor) to *E. coli* DH10B (recipient) has to be lower than the translocation of the Tn7714 transposon plus the conjugation of the new IncX plasmid in the *E. coli* SW4848. This result shows that the conjugation of the IncF plasmid depends on the donor-recipient combination.

The conjugation experiment with the transconjugant *E. coli* TSW4848-W1 indicates that the new IncX plasmid is capable of conjugating without any other plasmid (i.e., the IncF plasmid is not required for the new IncX plasmid to conjugate). Once the new IncX plasmid acquires the Tn7714 transposon, it is no longer cryptic, and it can be selected based on the ARGs coded in the transposon (aminoglycosides, sulfamethoxazole, tetracycline, and trimethoprim).

Tn7714 is a novel transposon of the Tn3 family that is a hybrid between Tn1721 and Tn21 (Fig. 3), i.e., it has two epitomes of the Tn3 family (18, 24). Tn21 and Tn1721 belong to the Tn21 clade (24), characterized for having highly conserved IRs (25). Our results showed that Tn7714 is a functional transposon that can translocate between different DNA molecules targeting different sites: five nucleotides in the IncF plasmid (5′-AATTA-3′) and six nucleotides in the new IncX plasmid (5′-TATGTC-3′; Fig. 1). Consistent with other reports of the transposases of the Tn3 family (24), the Tn7714 transposase used different IR (Fig. 3). A key characteristic of transposons of the Tn3 family is their translocation by replicative transposition in which, after transposition, the transposon leaves behind a copy (26). Therefore, the net result in the present work is an IncF plasmid with the original Tn7714 transposon and a new IncX plasmid with a copy of the Tn7714 transposon (Fig. 1C). Since the conjugation rate of the new IncX is higher than the IncF, the dispersion of the multidrug-resistant Tn7714 is enhanced.

Most public experimental data on transposons and conjugative plasmids are from bacteria collected from clinical settings (13, 27–29). Additionally, experimental works showing transposons’ movement in cloning vectors may not reflect what happens in conjugative plasmids found in the environment. Here, we present the first report of the relocation of a transposon between two plasmids in an environmental bacterium (Fig. 1). The translocation occurred from an IncF plasmid into a cryptic conjugative plasmid showing how cryptic conjugative plasmids are a significant concern because they can capture and disperse ARGs from the vast gene pool in the environments. The cryptic IncX plasmid described in this research exemplifies the vast collection of cryptic conjugative plasmids in the environment. This work augments our understanding of the mobilization of MGE in environmental bacteria. Results from this study show how cryptic conjugative plasmids interact with transposons to shape the horizontal transfer of ARGs, a phenomenon at the root of the current antibiotic-resistance crisis.

## MATERIALS AND METHODS

### Bacterial strains, growth media, and culture conditions

Bacteria were grown routinely at 35°C on solid media and at 35°C and 270 rpm in liquid media. We used Mueller-Hinton II broth (MHB; BD BBL, USA) and CHROMagar (CHROMagar Orientation, France) for routinely growing bacteria in liquid and solid media. We used Mueller-Hinton II agar (MHA; BD BBL, USA) to select bacteria in conjugation and transformation experiments.

*E. coli* DH10B (Invitrogen, Thermo Fisher Scientific, USA) and J53 were recipient strains during conjugation experiments (30, 31). *E. coli* SW4848 is an environmental isolate collected in the City Park Lake, Baton Rouge, LA (30°25'45" N and 91°10'2" W). *E. coli* SW4848 was isolated using selective media CHROMagar supplemented with 10 g/L lactose (Fisher Chemicals, USA), 5 g/L bile salts (Difco Bile Salts No. 3, Becton, Dickinson, and Company, USA), 4 g/L trimethoprim (MP Biomedicals, LLC, USA), and 76 g/L sulfamethoxazole (Sigma, USA). On this selective media, *E. coli* develops a pink color, and bile salts inhibit the growth of Gram-positive bacteria, facilitating the isolation of *E. coli*.

To isolate *E. coli* SW4848, a 150 mL lake water sample was passed through a 0.45 mm filter (GN-6 Metricel 47 mm S-Pack white gridded, Pall Corporation, USA). Then, the filters were placed on top of selective CHROMagar and incubated overnight at 35°C. A pink colony was streaked on fresh selective media the next day and incubated overnight. Finally, we used a single isolated colony to prepare glycerol stocks and biotyping using a MALDI-TOF mass spectrometer (Microflex, Bruker Daltonics GmbH and Co. KG, Germany). Bacterial strain stocks were prepared by storing bacteria grown overnight in MHB with glycerol (25% final concentration) at −80°C.

### Minimum inhibitory concentrations

During MICs, we followed the microdilution protocol described by the Clinical and Laboratory Standards Institute (32). We used *E. coli* ATCC 25922 (American Type Culture Collection[ATCC], USA) for quality assurance control (32). We tested streptomycin (Thermo Fisher Scientific, USA), sulfamethoxazole, tetracycline (Sigma, USA), and trimethoprim.

### Transformation and conjugation

We used *E. coli* DH10B electrocompetent cells prepared as indicated elsewhere (33) during transformation experiments. In addition, we transformed the competent cells with plasmids of *E. coli* SW4848 extracted with the QIAGEN Plasmid Midi Kit (Qiagen, Germany) following the manufacturer’s instructions. To select transformants, we used 16 mg/L of trimethoprim.

During conjugation using *E. coli* DH10B as the recipient, we used 100 mg/L sulfamethoxazole (Sigma) and 1,000 mg/L streptomycin for selecting transconjugants; note that transconjugants are selected by these antibiotics concentrations because the media contain a sulfamethoxazole concentration higher than the MIC of the recipient (but lower than the MIC of the donor) and a concentration of streptomycin higher than the MIC of the donor (but lower MIC than the recipient). As shown in Fig. 1A, the MIC of streptomycin for *E. coli* DH10 is 16,834 µg/mL, whereas for *E. coli* SW4848 is between 128 and 256 µg/mL.

When using *E. coli* J53 as the recipient, we selected transconjugants using 100 mg/L sulfamethoxazole and 100 mg/L sodium azide (Fisher Chemical, USA). In all the conjugation experiments, we supplemented the media with 100 mg/L Magenta-Gal (5-bromo-6-chloro-3-indoxyl β-D-galactopyranoside, CHEM-IMPEX, USA) to differentiate *E. coli* DH10B from other bacterial strains by color; while *E. coli* DH10B and its derivatives are cream, the rest of the *E. coli* strains are magenta.

We performed conjugations as follows: first, we grew 3 mL of donors and recipients overnight in 15 mL Falcon round-bottom tubes. Next, we adjusted the number of donors and recipients to a final volume of 1 mL using 0.85% NaCl in a 1.5 mL Eppendorf tube. During conjugations with *E. coli* SW4848 as the donor and *E. coli* DH10B as the recipient, we adjusted the cells to a final concentration of 1.5 OD_625_ donors and 0.5 OD_625_ recipients to increase the number of transconjugants. In the rest of the conjugations (as described in Fig. 2B and C), we adjusted the cells to a final concentration of 0.5 OD_625_ donors and 0.5 OD_625_ recipients. Then, we washed the cells once with 0.85% NaCl, resuspended the cells in 500 µL of 0.85% NaCl, and incubated the mating tube for 16 h. (overnight) at 35°C. To select DH10B transconjugants, we used sulfamethoxazole and streptomycin. For J53 transconjugants, we used sulfamethoxazole and sodium azide. Finally, we used sulfamethoxazole to select donors (in addition to the antibiotic, donors can be distinguished because they have a different color on selective agar). To calculate the conjugation frequency, we performed at least three independent experiments and divided the colony-forming units (CFU) of transconjugants by the CFU of donors (T/D).

### Plasmid analysis by electrophoresis

To compare plasmids between different strains, we extracted plasmids with phenol/chloroform/isoamyl alcohol (25:24:1, Fisher BioReagents, USA) as described elsewhere (34). We used a 25 cm long Sub-Cell GT Horizontal Electrophoresis System (Bio-Rad, USA) with 0.8% agarose gels (Fisher Scientific) at 130 V per 4 h for gel electrophoresis. After the electrophoresis, we stained the gel with 1 µg/mL ethidium bromide (Promega, USA) for 30 min and removed the excess ethidium bromide in water overnight. To compare the size of all plasmids studied, we ran all plasmids in a single gel shown in Fig. S1; to show in detail the size of large plasmids, we cropped this image to be shown in Fig. 1B and 2E.

### DNA library preparation and whole-genome sequencing

We grew bacteria overnight in 3 mL MHB and extracted their DNA using phenol-chloroform as described elsewhere (35). We used short reads (Illumina, USA) and long reads (Oxford Nanopore Technologies, UK) to perform whole-genome sequencing. An Illumina library was prepared with a Nextera Flex Library Prep, loaded into a High Output Flow Cell 300 cycles (2 × 150 bp paired-end reads), and run in a MiniSeq instrument with System Suite 2.0.0 (Illumina, San Diego, CA). A total of 1,132,724 Illumina reads were obtained for *E. coli* SW4848 and 1,395,924 Illumina reads for *E. coli* SW4848-W1. The long reads library was prepared with the Rapid Sequencing Kit (Oxford Nanopore Technologies [ONT]) and sequenced in a MinION. All the procedures were followed according to the manufacturer’s instructions. A total of 20,000 ONT reads (mean read length of 6,324 bp) were obtained for *E. coli* SW4848 and 17,920 ONT reads (mean read length of 16,233 bp) for *E. coli* SW4848-W1.

### *De novo* genome assembly and computational analysis

*E. coli* SW4848 and *E. coli* TSW4848-W1 genomes were assembled using Unicycler v0.4.8-beta (36). Default parameters were used for all software, including those for quality control of Illumina and ONT reads. No additional filters for Illumina and ONT reads were used before assembly. Genome coverage for *E. coli* SW4848 was 38.7× (30.2× with Illumina reads and 8.5× with ONT reads) and for *E. coli* TSW4848-W1 was 99.1× (42.6× with Illumina reads and 56.5× with ONT reads).

The following web services were used to annotate the genomes: PATRIC (37), MobileElementFinder (38), TnCentral (15), ISfinder (16), the Transposon Registry (39), and NCBI-BLAST. We performed the additional computational analysis with Geneious software. The program MSPlotter (40) made the alignment visualization shown in Fig. 3A. An in-house Python script plotted the conjugation frequency in Fig. 2E. Finally, all figures were customized for publication with Biorender.com.

## Data Availability

The genome sequences data were deposited in NCBI GenBank under BioProject PRJNA888808: BioSample SAMN31221298 for *E coli* SW4848 and SAMN31632844 for *E coli* TSW4848-W1.

## SUPPLEMENTAL MATERIAL

Figure S1 msphere.00252-24-s0001.tif Whole-plasmid gel electrophoresis analysis of the different strains shown in the present study.

ASM does not own the copyrights to Supplemental Material that may be linked to, or accessed through, an article. The authors have granted ASM a non-exclusive, world-wide license to publish the Supplemental Material files. Please contact the corresponding author directly for reuse.
