# Supplementary figures and images for: Cryptic environmental conjugative plasmid recruits a novel hybrid transposon resulting in a new plasmid with higher dispersion potential
Source: mSphere. 2024 May 21;9(6):e00252-24. doi: 10.1128/msphere.00252-24 (PMC11332342; doi:10.1128/msphere.00252-24)

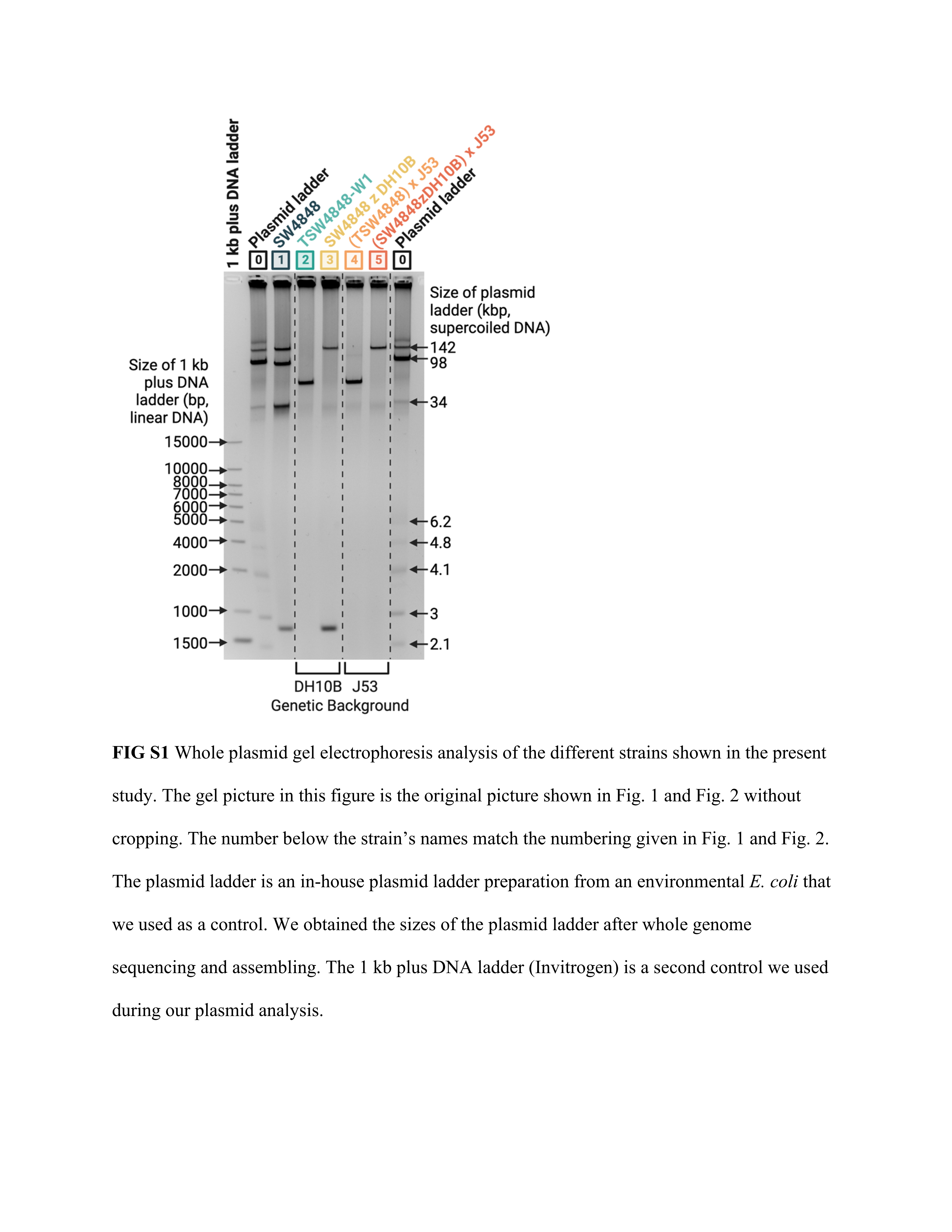

Supplement: Figure S1 — Whole-plasmid gel electrophoresis analysis of the different strains shown in the present study. [file msphere.00252-24-s0001.tif]
